# Zintl Phases: From Curiosities to Impactful Materials

**DOI:** 10.1021/acs.chemmater.3c01874

**Published:** 2023-09-04

**Authors:** Susan M. Kauzlarich

**Affiliations:** Department of Chemistry, University of California, One Shields Avenue, Davis, California 95616, United States

## Abstract

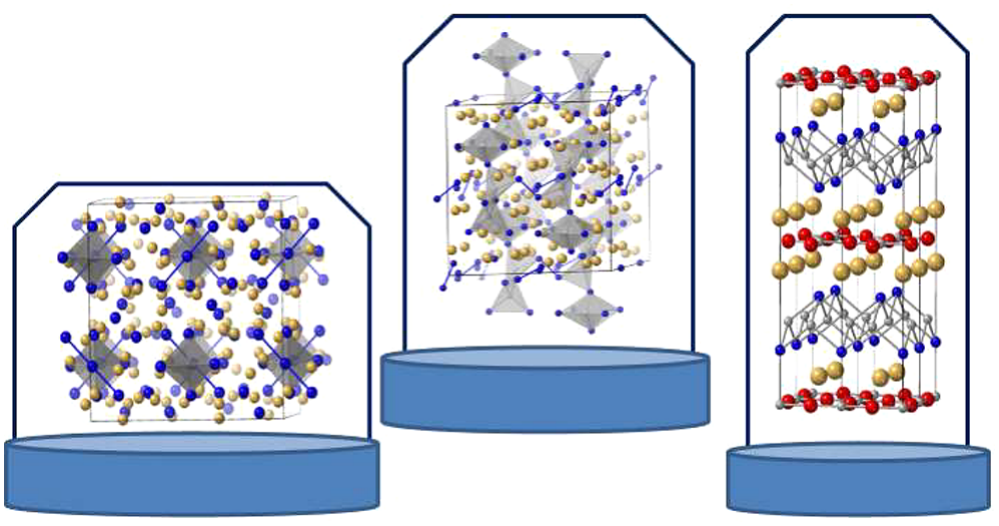

The synthesis of new compounds and crystal structures remains an important research endeavor in pursuing technologically relevant materials. The Zintl concept is a guidepost for the design of new functional solid-state compounds. Zintl phases are named in recognition of Eduard Zintl, a German chemist who first studied a subgroup of intermetallics prepared with electropositive metals combined with main-group metalloids from groups 13–15 in the 1930s. Unlike intermetallic compounds, where metallic bonding is the norm, Zintl phases exhibit a combination of ionic and covalent bonding and are typically semiconductors. Zintl phases provide a palette for iso- and aliovalent substitutions that can each contribute uniquely to the properties. Zintl electron-counting rules can be employed to interrogate a structure type and develop a foundation of structure–property relationships. Employing substitutional chemistry allows for the rational design of new Zintl compounds with technological properties, such as magnetoelectronics, thermoelectricity, and other energy storage and conversion capabilities. Discovering new structure types and compositions through this approach is also possible. The background on the strength and innovation of the Zintl concept and a few highlights of Zintl phases with promising thermoelectric properties in the context of structural and electronic design will be provided.

## Introduction

The synthesis of new compounds and crystal structures remains an extremely important research endeavor in pursuing technologically relevant materials. The ability to prepare new compounds with an entirely new, unforeseen structure or with properties predicted to be enhanced over a previously published variant is one of the exciting contributions of synthetic chemists. The balance of these two ideas (exploratory- and hypothesis-driven) toward synthesizing new compounds with technological applications can be considered high risk when first imagined but may result in significant technological advancement when combined with detailed characterization. The Zintl concept provides a rationale based on structure and bonding that can be employed in designing new compounds with exciting technological applications.^1−5^ Using the ideas of electron counting and chemical bonding, we can target specific structure types to test ideas for property outcomes and use Zintl counting rules to optimize the structure type further, providing a foundation of structure–property relationships. As highlighted below, Eduard Zintl pushed the boundaries of knowledge during his time by developing new synthetic tools and discovering new structures, employing detailed analytical and structural characterization. Similarly, with a broad view of solid-state materials chemistry, we initially developed methods to measure the properties of air-sensitive compounds to demonstrate the unique nature of transition-metal analogs of existing Zintl compounds with an eye toward potential applications,^6,7^ developed more air-stable analogs,^8^ and demonstrated these materials applications in the area of thermoelectricity.^9,10^ In addition to thermoelectrics, Zintl phases have the potential for applications in batteries,^11,12^ photovoltaics,^13^ catalysts,^14,15^ and hydrogen storage materials,^16^ to name a few.

Zintl phases are a subclass of intermetallics where the bonding can be described using simple chemical bonding principles such as ionic and covalent bonding interactions. Ionic bonding is associated with the electropositive cations that donate their electrons to the more electronegative elements to form extended polyanions or clusters with covalent or polar covalent bonding or isolated anions with their full octet.^2,3^ An important aspect of a Zintl phase is a well-defined relationship between the chemical and electronic structure, with the anionic component(s) satisfying valence bonding rules.^17^ This contrasts with Laves phases, which are intermetallics with the AB_2_ composition that crystallize in structure types such as Mg_2_Cu, Mg_2_Zn, and Mg_2_Ni, where the relative size of the elements and total electron count rather than chemical bonding considerations provide guiding principles.^18^ Care should always be taken when using any simple model, and electronic structure calculations provide a more detailed view of bonding.^18,19^ The strength of the Zintl approach, connecting chemical and electronic structure using valence bonding principles, allows for the rational design of new compounds with important technological properties, such as magnetoelectronics,^20−22^ thermoelectricity,^10,23−25^ and other energy storage and conversion capabilities.^26−29^ Another area of research where Zintl phases are of great importance is in the synthesis of new materials that are difficult to prepare by bottom-up solution reaction processes.^5^ Metathesis reactions of binary or ternary Zintl phases with metal halides result in a variety of new materials. Zintl phases can be employed as precursors to novel films,^30^ nanomaterials, and 2D materials.^31−34^ Eduard Zintl initially investigated ammonia solutions of alkali metals with heavy main-group metalloids, such as Pb, Bi, and Sb, to understand the color changes and determine the composition, as described below.

## What’s in a Name?

Zintl phases are a subset of intermetallics named after Eduard Zintl (1898–1941), born on January 21, 1898, in Weiden in Oberpfalz, Bavaria;^2^ this year would be his 125th birthday. His education began in Weiden and Bayreuth; he completed his high school diploma in Munich when his family moved there. He was drafted into the German military during World War I, after which he started his studies in chemistry at the Bavarian Academy of the Sciences in Munich at the age of 21. He began his thesis work in the laboratory of Otto Hönigschmid, head of the German Atomic Weight Laboratory. The topic of Zintl’s dissertation, which he completed at age 25, was the first atomic weight determination of some elements. In independent research, he developed methods of potentiometric titration for quantitative analysis. He published his dissertation work and a comprehensive textbook, *Introduction to the Study of Inorganic Chemistry* (Enke Publisher, 1923), with the sentence in the foreword, “Modern inorganic chemistry is applied physical chemistry.” ^35^ This statement highlights Zintl’s interest in bringing physical methods to the forefront of inorganic chemistry.

Eduard Zintl took a position as an associate professor at the University of Freiburg in Breisgau at the age of 29, where he started his work on what would be eventually called Zintl phases.^36^ X-ray diffraction was a new technique that he added to his suite of experimental methods. To succeed in these efforts, he developed methods for preparing and handling air-sensitive products and determining their powder diffraction.^35^ He worked out which phases crystallized in saltlike as opposed to metallic structures. He reported two new structure types, NaTl and NaZn_13_, and showed that the saltlike structures corresponded to already-known oxides and halides.^37,38^ He was also fascinated by early reports concerning colored solutions of alkali-metal metalloids when dissolved in liquid ammonia.^31,39,40^ This led to his publications on Zintl anions such as Pb_9_^4–^, Sn_9_^4–^, Sb_7_^3–^, and As_7_^3–^, identifying their stoichiometry and charge by potentiometric titration.^31,39−41^

**Figure 1:**
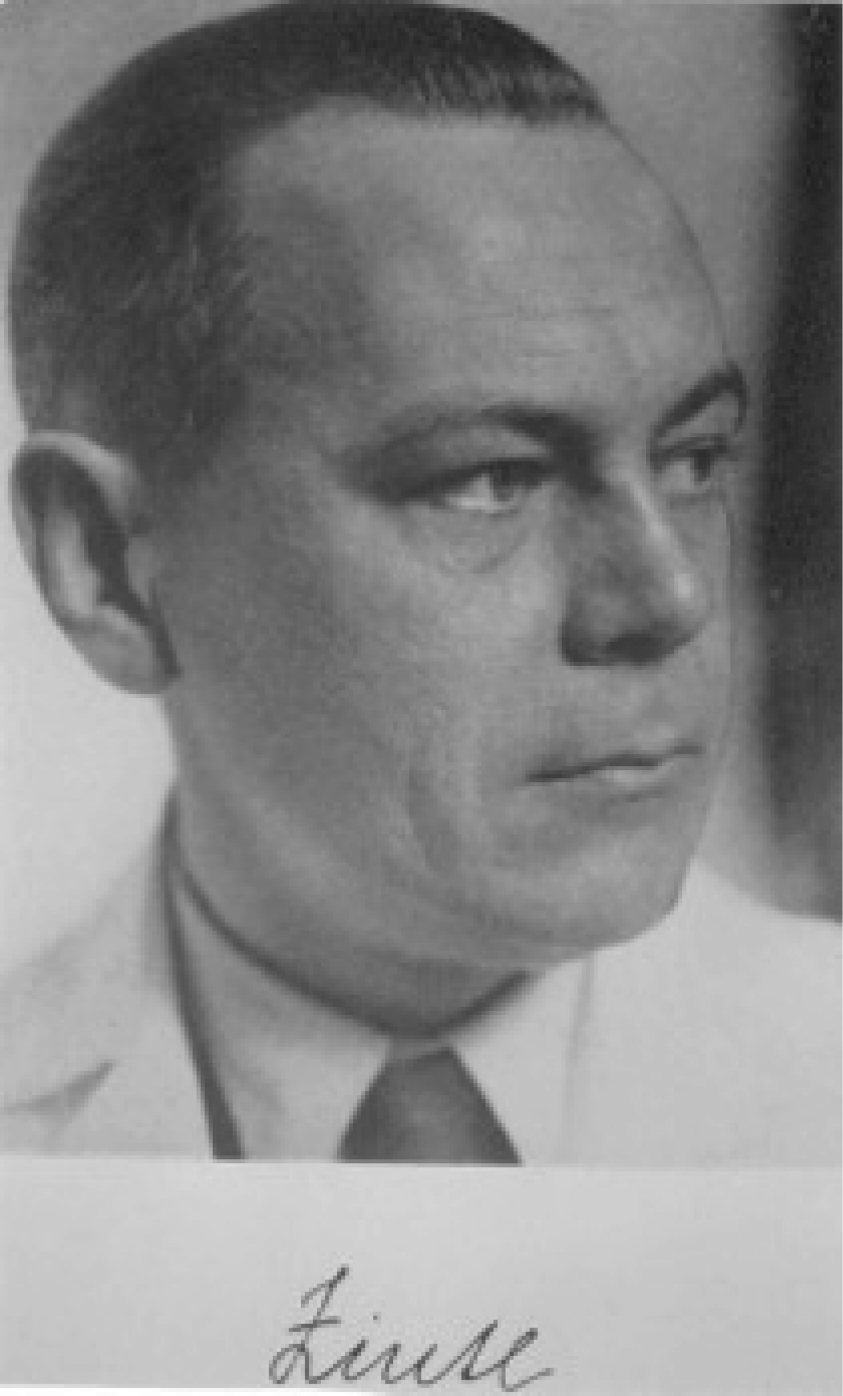
Eduard Zintl at Darmstadt University of Technology. Used with permission of Elsevier, from *Chemistry, Structure, and Bonding of Zintl Phases and Ions*; Kauzlarich, S. M., Ed.; VCH Publishers, Inc.: New York, 1996. Permission conveyed through Copyright Clearance Center, Inc.

Eduard Zintl, shown in Figure 1, was appointed a full professor of chemistry and head of the Institute for Inorganic Chemistry at the Technical University of Darmstadt in 1933. Building on his development of potentiometric analysis of alloys in ammonia, he developed procedures to handle air-sensitive products for X-ray diffraction analysis. He connected his structural work on the compounds AM_13_ (A = Ca, Sr, Ba, K, Rb, Cs; M = Zn, Cd) to analogous silicates where the metal was a framework structure in which the cation resided. Wilhelm Klemm further articulated this idea concerning the NaTl structure, which can be described as a double diamond structure, with Na and Tl forming interpenetrating diamond substructures, rationalized from Na donating an electron to Tl, forming a Tl^–^ anion with 4 valence electrons, like a group 14 element. The Tl^–^ anion behaves as a pseudo-group 14 element with 4 covalent bonds and adopts the diamond structure with each Na^+^ cation as charge-balancing and space-filling. Therefore, the general idea of bonding in these intermetallic phases is referred to as the Zintl or Zintl–Klemm concept.^18,19^ Two major advancements of Zintl’s research were the determination of which elements can form anions with non-noble metals and the idea of a border between compounds of saltlike structures versus metallic compounds (referred to as the Zintl border). The progression from saltlike (ionic) to covalently bonded structures had already been documented for halogens and chalcogens; Zintl’s work showed the transitional area between ionic or saltlike compounds and metallic phases. He expanded from alkali metals to alkaline earths and elements of zinc and copper groups. The Liebig commemorative medal recognized his achievements in 1938. He also became the editor of the *Zeitschrift für Anorganische und Allgemeine Chemie* and the vice-president of the Verein Deutscher Chemiker. Eduard Zintl died in 1941 and did not move into the nearly finished new institute at Darmstadt posthumously named in his honor: The Zintl Institute for Inorganic and Physical Chemistry. His contribution to the design of the building emphasized the connection between teaching and research and became a symbol of the state of inorganic chemistry.^42,43^

The Zintl or Zintl–Klemm concept, where an electropositive metal donates its electrons to the more electronegative metals, which can be described by a combination of ionic and covalent bonding, does an effective job of rationalizing the structure of Zintl phases.^18,44^ Combining various metallic elements gives rise to compounds with exact compositions (or narrow homogeneity ranges) and semiconducting properties through ionic and covalent bonding interactions. Many new Zintl phases, including those with complex anions, were discovered in the years following Zintl’s death.^1,3,45,46^

## Transition-Metal-Containing Zintl Phases

Our interest in designing new compounds to provide unusual magnetic and electronic properties started with considering ternary Zintl phases as a starting framework to transition from main-group metalloids to transition-metal-containing phases. There were many main-group Zintl compounds whose structures had been characterized and contained isolated clusters, 3D networks, 2D layers, or 1D chains, suggesting that a simple replacement of the main-group element with a transition metal would lead to unusual and exciting magnetic and electronic properties. In addition, since many of these Zintl structure types could be prepared with the group 15 (pnictogen) series from P to Bi, one could transverse from semiconductor to metallic behavior by simply changing the identity of the pnictogen and therefore the bonding from highly directional covalent to delocalized or metallic bonding. Inspired by the ideas and reviews of the Darmstadt group of H. Schäfer on Zintl phases, where a few examples of Zintl phases containing transition metals from the far right of the *d* block, such as Cu and Zn, and the far left, such as Ti and Hf,^2,3^ were provided, a natural extension was to consider transition metals from the middle of the *d* block.

**Figure 2:**
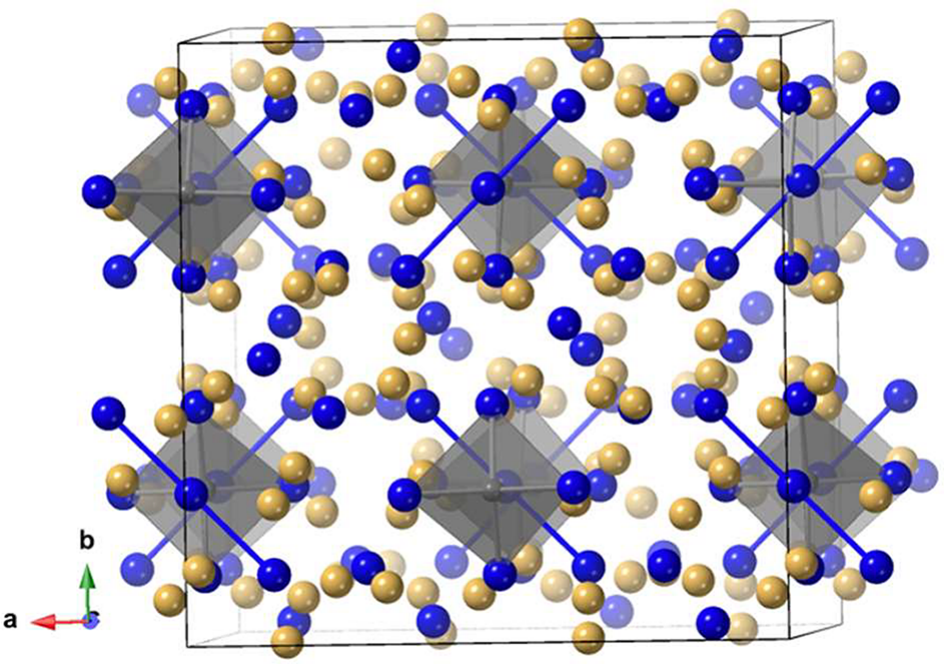
View of Ca_14_MnBi_11_ along the *c* axis. Ca atoms are shown in gold, Mn in gray, and Bi in blue. The MnBi_4_ translucent tetrahedra and Bi_3_ linear anions are indicated.

This direction of research resulted in the discovery of a transition-metal analog of the Zintl phase, Ca_14_AlSb_11_.^47^ Ca_14_MnBi_11_, the structure of which is shown in Figure 2,^6^ is isostructural to Ca_14_AlSb_11_. The Zintl analysis of the structure of Ca_14_AlSb_11_ was explained with AlSb_4_ being formally a 9– anionic cluster isosteric to AlO_4_^5–^ with the O^2–^ ions substituted by Sb^3–^.^2,47^ The linear group was postulated to be isosteric to XeF_2_, with a 3-centered-4-electron bonding motif and the remaining anions being Sb^3–^. This gives rise to the description of the formula unit as consisting of 14Ca^2+^ + AlSb_4_^9–^ + 4Sb^3–^ + Sb_3_^7–^.

Electronic structure calculations have validated this description of the Ca_14_MPn_11_ main-group structure.^48,49^ In the first example of the transition-metal-containing phase, Ca_14_MnBi_11_, it was presumed that Mn should be isoelectronic to Al and, therefore, 3+. The magnetic and electronic properties of this phase and other analogs were studied in detail and supported this hypothesis with a compressed tetrahedron of MnBi_4_ and a magnetic moment of about 4 spins (4 μ_B_).^4,7,50,51^ As more transition-metal compounds (Zn and Cd analogs) were added to this structure type, whether these compounds could be considered Zintl phases was called into question.^52^ In addition, the unique magnetic and electronic properties required additional validation in addition to the simple Zintl rules. For example, the A_14_MnPn_11_ (A = Ca, Sr, Ba; Pn = Sb, Bi) compounds showed unique magnetic and electronic properties.^8,50,53−56^ Ca- and Sr-containing A_14_MnBi_11_ compounds were ferromagnetic, with Curie temperatures as high as 55 K, while the Ba analog was antiferromagnetic, with a Néel temperature of 15 K. Electronic structure calculations showed that, while these Mn-containing phases showed a band gap like a semiconductor, the valence band was one electron short of being filled and the oxidation state of Mn in the MnBi_4_ cluster was Mn^2+^.^57^ The electronic structure calculations showed that Ca_14_MnBi_11_ was nearly a half-metallic ferromagnet with a localized hole in the tetrahedral MnBi_4_ tetrahedral unit; this hole partially compensates for the high-spin *d*^5^ Mn^2+^ moment, resulting in the spin density of 4 μ_B_, consistent with experiment. In accordance with the Zintl concept, one can describe Ca_14_MnBi_11_ and the structural analogs of this phase as p-type semiconductors with carrier concentration equivalent to a hole.^57,58^

By adding transition metals to prepare isostructural compounds, serendipity occasionally intervened to produce new structure types that became the starting point for further investigations, such as Sr_21_Mn_4_Sb_18_ and the layered suboxide Ba_2_Mn_3_P_2_O_2_.^59,60^

**Figure 3:**
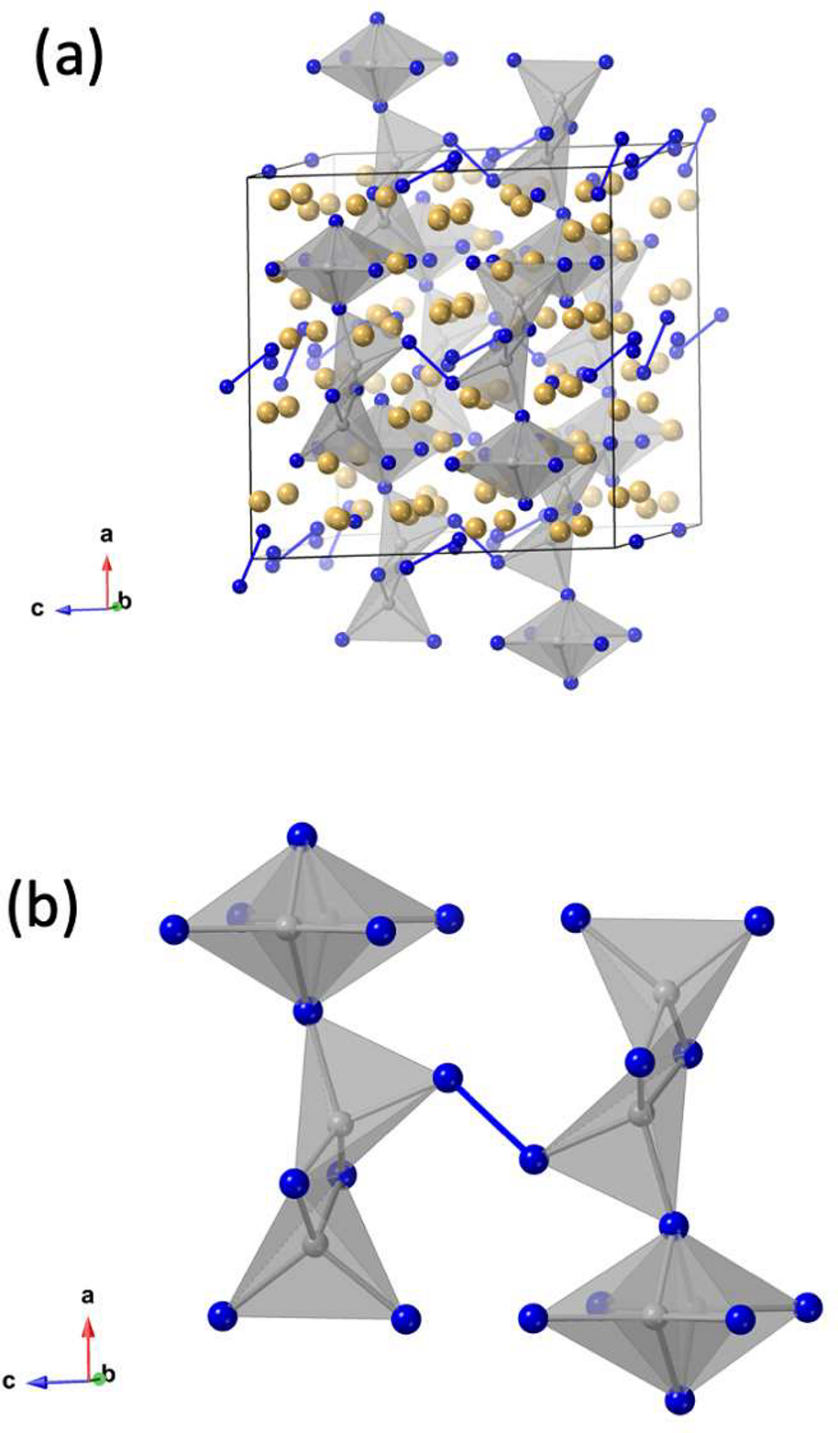
Views of (a) the unit cell of Sr_21_Mn_4_Sb_18_, with the MnSb_4_ translucent tetrahedra and Sb_2_ anions indicated, and (b) the [Mn_8_Sb_22_]^48–^ cluster. Sr atoms are shown in gold, Mn in gray, and Sb in blue. The MnSb_4_ translucent tetrahedra are highlighted.

**Figure 4:**
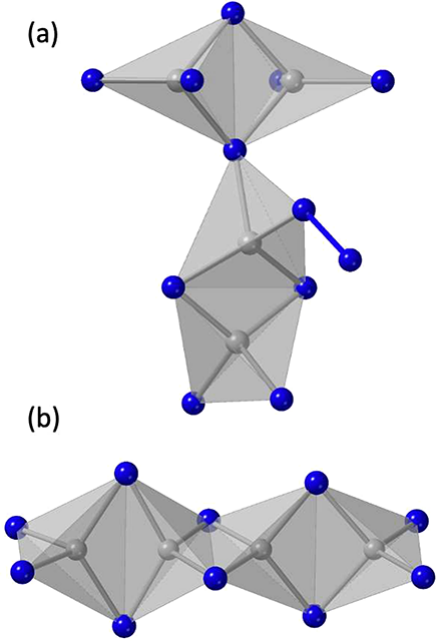
Clusters of (a) the orthorhombic *Cmce* and (b) monoclinic *C*2/*c* space groups of compositions of A_21_M_4_Pn_18_. The M atoms are shown in gray, with the tetrahedral coordination indicated and Pn in blue.

Sr_21_Mn_4_Sb_18_ (Figure 3) was the first compound of this composition, crystallizing in the monoclinic *C*2/*m* space group, and the idea was further expanded into a large number of structure types with the general composition A_21_M_4_Pn_18_, where A = Ca, Sr, Ba; M = Mn, Zn, Cd; and Pn = As, Sb, Bi.^61−63^ Compounds of this composition have not been systematically investigated but crystallize in three structure types reported to date: orthorhombic *Cmce*, monoclinic *C*2/*m*, and monoclinic *C*2/*c*.^25^ To date, the only arsenide is Ca_21_Zn_4_As_18_.^63^ All compounds with these compositions comprise MPn_4_ tetrahedra that are corner- or edge-shared to give small clusters, and their structures can be described as consisting of 21A^2+^ + [Mn_4_Pn_14_]^34–^ + 2[Pn_2_]^4–^. The cluster for the *C*2/*m* space group represented by the Sr_21_Mn_4_Sb_18_ structure type is shown in Figure 3(b). The clusters for the Ba_21_Cd_4_Sb_18_ structure type,^64^ with the orthorhombic *Cmce* space group and monoclinic *C*2/*c* of α-Ca_21_Mn_4_Sb_18_,^61^ are shown in Figure 4. The structures show significant complexity, and the potential for chemical substitutions with atoms similar in size and bonding (either isovalent or aliovalent) provides a large composition space for property optimization. The magnetic properties of these compounds need to be better understood. Sr_21_Mn_4_Sb_18_ shows temperature-independent paramagnetism above 80 K, with possible spin fluctuations below 12 K. Properties of the Ca and Sb analog have not been reported; however, electronic calculations on Ca_21_Mn_4_Bi_18_ suggested a small-bandgap semiconductor with antiferromagnetic coupling.^62^

**Figure 5:**
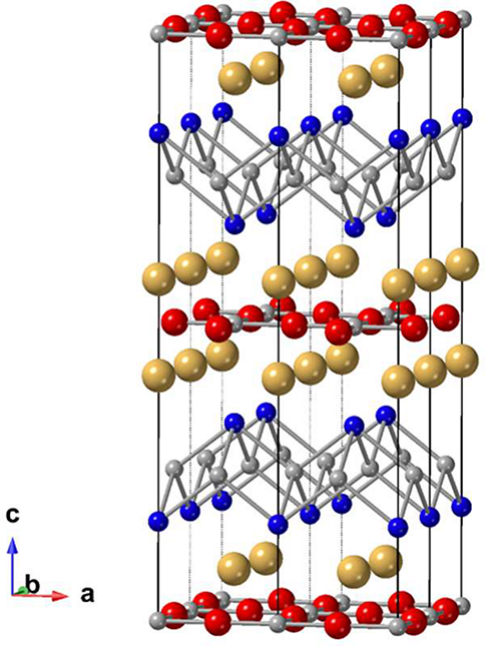
View of Ba_2_Mn_3_Sb_2_O showing the MnO_2/2_ and Mn_2_Pn_2_ layers. Ba is in gold, Mn in gray, Sb in blue, and O in red.

The layered pnictide oxide compounds, A_2_Mn_3_Pn_2_O (A = Sr, Ba; Pn = As, Sb, Bi), shown in Figure 5, were originally reported by H. Schäfer’s group^65^ and presented in a review article.^2^ The structure received great interest and stimulated our interest in synthesizing layered pnictide oxides containing Mn and Ti.^60,66−75^ The layered suboxide compounds that the Kauzlarich group has investigated have been reviewed^76−79^ and laid the groundwork for many other investigations into these structure types. Again, the possibilities of alio- and isovalent substitutions have led to superconductivity^80^ and other exotic magnetic properties.^81,82^

New compositions and structures expanded the Zintl concept to include transition metals through isostructural relationships, and the Zintl concept is surprisingly robust in rationalizing those new structures with no main-group-containing Zintl analog.^4−6,17−19,25,44,83−87^

## Rare-Earth Zintl Phases

The replacement of alkali metals with Yb and Eu rare-earth cations provided an exciting new research direction and even more spectacular magnetic and electronic properties, including colossal magnetoresistance.^88−93^ The term “colossal magnetoresistance” is employed to describe the strong correlation between electrical resistance and magnetic field, typically by orders of magnitude, and mostly observed in manganese oxide materials.^94^ The replacement of the electropositive alkaline-earth cations in a Zintl phase with the 2+ rare-earth cations, Yb and Eu, resulted in isostructural phases that were less susceptible to oxidation.

In the example of compounds from the Ca_14_AlSb_11_ structure type, the addition of rare-earth cations has allowed for more property measurements and paved the way for measuring thermoelectric properties. X-ray magnetic circular dichroism (XMCD) on single-crystal Yb_14_MnSb_11_^93,95^ further supported the electronic structure calculation findings presented above.^57^ The current understanding of the magnetic and electronic data is consistent with all the transition metals being in the 2+ oxidation state and Yb can be 2+, intermediate valency, or a mixture of 2+/3+, depending on the identity of the transition or main group metal in the tetrahedron.^57,96−98^ All of the metal-containing compounds can be considered Zintl phases. As such, each satisfies the valence rules in a slightly different fashion. Yb_14_MnSb_11_ is composed of 14 Yb^2+^ cations that donate electrons to a (MnSb_4_)^9–^ tetrahedron, a linear anion, Sb_3_^7–^, and 4 isolated Sb^3–^ anions. Mn is shown to be Mn^2+^, with the tetrahedral cluster considered to contain the *d*^5^ + hole electronic configuration, resulting in ∼4 unpaired electrons in the ferromagnetically ordered phase.^97,99^ Yb_14_MgSb_11_ has a mixture of about 13 Yb^2+^ and 1 Yb^3+^ donating electrons to a (MgSb_4_)^10–^ tetrahedron, a linear anion, and 4 isolated Sb^3–^ anions.^97,100^ Yb_14_ZnSb_11_ has intermediate valency for Yb, with an average oxidation state of +2.07.^97,101^ Colossal magnetoresistance and other unique magnetic properties have been measured in the Yb and Eu analogs of the Mn-containing phase.^21,88,91,102−106^

Transition-metal analogs isostructural to the alkaline-earth phases are found in Yb_21_Mn_4_Sb_18_.^107^ This phase comprises 21 Yb^2+^ cations that donate electrons to a linear (Mn_4_Sb_10_)^22–^ anion, 2 Sb_2_^4–^ anions, and 4 Sb^3–^ anions. We showed that the structure was complex, and electronic structure calculations revealed that the Mn_4_Sb_10_ chain mostly contributed to the states near the Fermi level. Preliminary studies on isovalent substitutions suggest that further research would be promising.^108^ In addition, there are a number of Eu-containing analogs that are worth investigating further.^25^

## Zintl Phases for Thermoelectric Applications

Thermoelectric materials can harvest energy by converting a temperature gradient into electricity. This unique ability has many applications in industry, including the use of Peltier coolers for refrigeration technology and waste heat recovery to generate power.^109,110^ There have been several reviews of Zintl phases and thermoelectric applications;^22,29,111^ therefore, in this Perspective, I will be brief on this last point. The generally accepted materials to explore for optimal thermoelectric applications are identified as semiconducting compounds with structural complexity that can be doped or substituted with isovalent or aliovalent atoms to increase or reduce the carrier concentration. This makes Zintl phases prime candidates as thermoelectric materials, as one can target ionically bonded cations or covalently bonded metal/metalloid–pnictide networks for isovalent or aliovalent substitutions to tune the electronics and carrier concentration.

The thermoelectric community is mostly focused on improving the dimensionless figure of merit (*zT*), which combines thermal conductivity, κ (W/m K), electrical resistivity, ρ (Ω-m), the Seebeck coefficient, *S* (V/K), and absolute temperature, *T* (K), according to *zT* = . The higher the *zT* value, the better the thermoelectric conversion efficiency of a temperature gradient into electricity or vice versa at a particular temperature. The thermal conductivity, electrical resistivity, and Seebeck coefficient depend on carrier concentration discordantly, making optimization of a material difficult.^112^ However, low lattice thermal conductivity naturally arises from a large unit cell or anharmonic vibrational modes, thereby making it relatively straight forward to start with a structure type that has a complex unit cell or has structural features such as covalently bonded layers with ionically bonded cations. One can propose whether the compound should be a semiconductor by employing the Zintl concept and counting electrons. Finally, the most difficult prediction is the magnitude of the Seebeck coefficient.^112^ Considering the various applications of orbital contributions to a high density of states at the Fermi level with high degeneracy provides a simplistic starting place for the search for structure types in the design of new compounds.

Once a material’s thermoelectric properties are measured, its thermoelectric performance can be optimized by systematically changing the carrier concentration. The combination of ionic and covalent or polar-covalent bonding found in Zintl phases makes them an ideal group for testing structure–property correlations and requirements for optimal thermoelectric performance.^10,23^ Noticing that many of these Zintl phases met the requirements for thermoelectric applications, we initiated an investigation of thermoelectric transport properties starting with Yb_14_MnSb_11_.^9,21^ This compound was initially chosen because we expected it to have low thermal conductivity because of the large unit cell; we knew that it had fairly low electrical resistivity and showed magnetoresistive behavior. We also knew that it had a high melting point. Therefore, this material was a good starting place in terms of structure to expect a high-temperature, high-efficiency thermoelectric material. Compounds of the general formula Yb_14_MSb_11_ (M = Mn, Mg, Zn)^113−115^ are some of the most efficient high-temperature p-type thermoelectric materials. Finding efficient materials in this temperature range is important due to the greater device efficiencies that result from large thermal gradients. This is especially true for p-type materials, whose thermoelectric efficiencies are lower than those of high-temperature n-type materials. In addition to this structure type, we have shown high thermoelectric efficiency in other Zintl phase materials.^107,108,116,117^

Aside from our contributions to research on Zintl phases for thermoelectric applications, there have been many other contributions to the field. Many Zintl phases have been shown to exhibit excellent thermoelectric properties. Zintl phases will remain important for thermoelectric applications as they compete with and surpass some historical state-of-the-art materials. New insights into the synthesis of these complex phases, property measurements, defect chemistry, and substitution chemistry will impact our understanding and guide the development of even better materials for direct thermal-to-electrical energy conversion. Additionally, their crystal and electronic structures provide a good beginning for developing materials for various other applications mentioned herein.
